# Implementation of a Nutrition Program Reduced Post-Discharge Growth Restriction in Thai Very Low Birth Weight Preterm Infants

**DOI:** 10.3390/nu8120820

**Published:** 2016-12-17

**Authors:** Suchada Japakasetr, Chutima Sirikulchayanonta, Umaporn Suthutvoravut, Busba Chindavijak, Masaharu Kagawa, Somjai Nokdee

**Affiliations:** 1 Department of Nutrition, Faculty of Public Health, Mahidol University, Bangkok 10400, Thailand; scdj01@yahoo.com; 2 College of Medicine, Rangsit University, Bangkok 10400, Thailand; 3 Department of Pediatrics, Faculty of Medicine, Ramathibodi Hospital, Mahidol University, Bangkok 10400, Thailand; u_suthut@yahoo.com; 4 Department of Pharmacy, Faculty of Pharmacy, Mahidol University, Bangkok 10400, Thailand; pybcd@mahidol.ac.th; 5 Institute of Nutrition Sciences, Kagawa Nutrition University, Saitama Prefecture 350-0288, Japan; mskagawa@eiyo.ac.jp; 6 Nursing Department, Buddhasothorn Hospital, Chachoengsao 24000, Thailand; somjainokdee@gmail.com

**Keywords:** very low birth weight preterm infant, growth restriction, post-discharge, nutrition program

## Abstract

Very low birth weight (VLBW) preterm infants are vulnerable to growth restriction after discharge due to cumulative protein and energy deficits during their hospital stay and early post-discharge period. The current study evaluated the effectiveness of the preterm infant, post-discharge nutrition (PIN) program to reduce post-discharge growth restriction in Thai VLBW preterm infants. A prospective, non-randomized interventional cohort study was undertaken to assess the growth of 22 VLBW preterm infants who received the PIN program and compared them with 22 VLBW preterm infants who received conventional nutrition services. Infant’s growth was recorded monthly until the infants reached six months’ corrected age (6-moCA). Intervention infants had significantly greater body weights (*p* = 0.013) and head circumferences (*p* = 0.009). Also, a greater proportion of the intervention group recovered their weight to the standard weight at 4-moCA (*p* = 0.027) and at 6-moCA (*p* = 0.007) and their head circumference to the standard head circumference at 6-moCA (*p* = 0.004) compared to their historical comparison counterparts. Enlistment in the PIN program thus resulted in significantly reduced post-discharge growth restriction in VLBW preterm infants. Further research on longer term effects of the program on infant’s growth and development is warranted.

## 1. Introduction

Very low birth weight (VLBW) preterm infants are those born before 37 weeks gestation weighing less than 1500 g, and they comprise between 4% and 8% of neonatal live-births [1,2]. Survival of these small infants has significantly improved mainly due to the advancement of medical intervention in neonatal intensive care units (NICU) [2,3]. However, the extra-uterine growth restriction (EUGR), defined as a decrease in *z*-score greater than two standard deviations (SD) between birth to discharge [4], is common in the preterm population [5]. A recent cohort study estimated that a prevalence of being small for gestational age (SGA) among VLBW infants (birth weight 750–1500 g) born between 2005 and 2012 to be 44.4%–58.8% at discharge [6]. This may be caused by several health problems such as need for respiratory support, unstable glucose levels, early sepsis, risk of necrotizing enterocolitis and feeding intolerance during NICU stay. These concerns impact highly on the prescribed feeding regimen and result in unrecovered early protein and energy deficits accumulated during hospital admission [7]. Moreover, post-discharge growth restriction also usually occurs in these infants at 28%–40% until 18–22 months corrected age (moCA) [8]. These poor postnatal growths are potentially associated with developmental delay [8], obesity and related complications, such as cardiovascular disease, type II diabetes mellitus and hypertension during adulthood [4,8,9].

In order to compensate for these nutritional deficits and improve the EUGR, the initiation of aggressive parenteral nutrition (PN) in the first week of life and fortified human milk (HM) to this population has been suggested during their hospital stay [10,11,12]. Furthermore, enriching HM or formula is widely recommended to decrease post-discharge growth restriction and accelerate catch-up growth [10,13]. However, the HM feeding rate in preterm infants usually declines after discharge [14]. Enriched-nutrient formula (EF) is therefore supplemented to provide adequate intakes of energy and nutrients to meet the infant’s needs [15,16,17].

In 2012, a preliminary study was performed at Buddhasothorn Hospital, Chachoengsao, Thailand. It investigated the nutritional care services and growth outcomes in 85 VLBW preterm infants at pre- and post-discharge. The findings revealed that the HM feeding rate was decreased from 87% at discharge to 7% at 6-moCA. After discharge, these infants received unfortified HM and/or standard term formula (TF). Moreover, 85% of VLBW preterm infants were below the standard growth at discharge and 43% remained as below the standard growth at 6-moCA.

In order to reduce post-discharge growth restriction in this population, the preterm infant, post-discharge nutrition (PIN) program was developed and implemented at Buddhasothorn hospital in early 2014. Based on recent recommendations [10,15,18], it focuses on providing an EF in addition to fortified HM with regular monitoring after discharge to ensure infant nutritional intakes. The aim of the present study was to compare growth outcomes in VLBW preterm infants who enlist in the PIN program with those who received conventional nutrition services.

## 2. Materials and Methods

A prospective non-randomized interventional cohort study was conducted at Buddhasothorn Hospital, located in Chachoengsao province, Thailand. The present study was approved by the Institutional Review Boards of the Faculty of Public Health, Mahidol University (COA. No. MUPH 2014-002), Buddhasothorn Hospital (COA. No.EC-CA 007/2556) and Chachoengsao provincial health office (COA. No. PH_CCO_REC 003/56). The current study used the subgroup of the study that registered as a clinical trial with the Thai clinical trials registration number of TCTR20160211001.

### 2.1. Participants

A total of 22 VLBW preterm infants who were admitted and discharged from the NICU between March 2014 and February 2015 and given the PIN program were invited to participate in the current study. Detailed verbal and written explanations were given to parents of VLBW preterm infants and informed written consent was obtained from the parents of each participant. These infants were compared with records of 22 VLBW preterm infants who were born and discharged from the NICU between January 2012 and December 2013. The intervention group was enlisted in the PIN program after discharge to 6-moCA whereas; the comparison group received a conventional nutrition service at post-discharge for the same period of time.

A sample size of 22 preterm infants for each group was determined based on a previous study using a power of 80% and a significance level of 5% to detect a program effect of an increase of 5% on the body weight with favorable outcomes at 6-moCA [19]. Inclusion criteria of the study include: (1) birth at ≤34 weeks gestation; (2) birth weight ranged between 750 and 1499 g; (3) no use of any medical feeding tube at discharge; and (4) residence in Chachoengsao. On the other hand, infants were excluded if they had: (1) severe respiratory problems; (2) serious congenital anomalies; (3) more than or equal to grade III periventricular or intraventricular hemorrhage; (4) surgical necrotizing enterocolitis; (5) gastrointestinal perforation; (6) severe retinopathy of prematurity; (7) severe birth asphyxia [6,19]; and also if (8) the mothers cannot communicate with researchers.

### 2.2. Recruitment Process of the Participants

**Figure 1:**
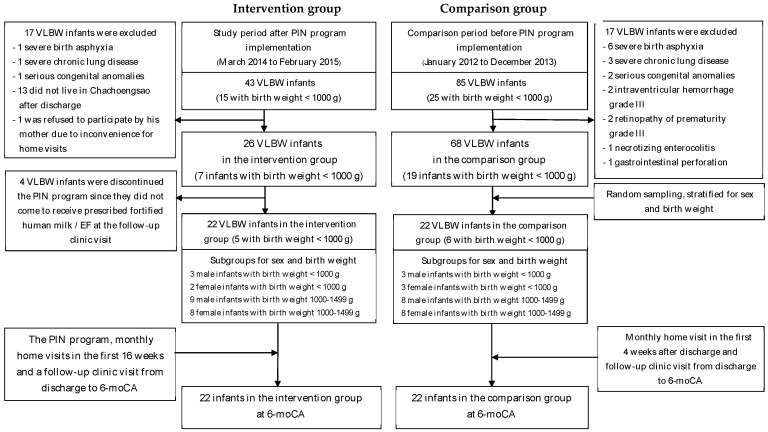
Recruitment process of participants. VLBW = very low birth weight, PIN = preterm infant post-discharge nutrition, EF = enriched-nutrient formula, g = gram, moCA = months corrected age.

Figure 1 describes the recruitment process of 22 VLBW preterm infants for both intervention and comparison group. The infants born in the comparison period were randomly assigned to the historical comparison group, stratified for sex and birth weight.

### 2.3. Nutritional Strategies Conducted for Both Groups during Hospital Admission

Until their discharge from hospital, both groups received parenteral and enteral nutrition. Infants in both groups received individualized PN formulation in the first few weeks of life. When the infant’s clinical status was improved, trophic oral feeding was introduced until the full feedings of at least 120–150 mL/kg/day were achieved [20]. All preterm infants mainly received their mother’s own milk. Mothers were instructed by NICU nurses to assess volume intake of breast milk by test-weighing procedure [21] before discharge. In order to increase energy density from 20 to 24 or 27 kcal/oz during NICU stay as recommended by recent studies [22,23,24], infants were given fortified HM that was prepared by adding preterm formula (PF) into expressed HM (calculated by definite formula) once their volume of feeding reached 100 mL/kg/day [20,25].

### 2.4. The PIN Program Conducted for the Intervention Group

The PIN program is a post-discharge nutritional plan of feeding fortified HM and/or EF for VLBW preterm infants from discharge to 6-moCA. It is based on a commentary by the European Society for Pediatric Gastroenterology, Hepatology, and Nutrition (ESPGHAN) committee on nutrition [22] and a nutrition care practice guideline developed by the Oregon Pediatric Nutrition Practice Group (OPNPG) [15]. The program was developed based on outcomes from focused group discussions of a multi-disciplinary team in the hospital. Four experts—including two pediatricians, one neonatologist, and one nurse—reviewed and approved the program. It was implemented as a routine nutritional service at Buddhasothorn hospital since January 2014. The program also includes education on mothers prior to their discharge such as benefits of breastfeeding to preterm infants [26], essential knowledge for lactating mothers [27,28], preparation method of fortified HM, feeding practices [29,30], and personal hygiene for their infant care after hospital discharge [29,30]. The contents of the education program were based on previous studies that recommended the importance of these variables [31,32].

#### 2.4.1. Enriched Milk Intake

The fortified HM suggested in the program was prepared by adding a given calibrated spoon (0.42 g, contains 2.1 kcal) of a powdered preterm formula (PF) (Pre-Nan Abott, Bangkok, Thailand) per oz (30 cc) of expressed HM to prepare milk with energy density of 22 kcal/oz. All intervention infants received fortified HM at discharge. If its volume was less than 50% of daily total volume intake, EF (Enfalac catch up care, Mead Johnson, Bangkok, Thailand) was supplemented to meet the infant needs with a goal of energy including 85–120 kcal/kg/day and 1.5–2.5 g/kg/day of protein according to their corrected age [10,15,22].

Specific written instructions on the fortification of HM and reconstitution of EF powder as well as containers for the preparation and storage of reconstituted milk were provided to each parent. In addition, the written instructions were repeated verbally and parental understanding was confirmed by research personnel. Dietary intake records were given to parents at discharge to assess actual milk and complementary food intake of infants. At the follow-up clinic, the mothers or caregivers were asked for more detailed information of their records. Volume of fortified expressed HM, EF, and other fluid intakes were determined by measuring in oz according to the label on the milk bottle.

#### 2.4.2. Introduction of Semi-Solid Foods

The introduction of semi-solid foods was commenced if: (1) they reached 4–6 months after birth; (2) body weight was not less than 5 kg; (3) they had no extrusion reflex; (4) they had well controlled neck and chair sitting posture; (5) they developed an ability to receive spoon feeding; and (6) they developed an ability to show desire or refusal of food [33,34,35,36]. Type and formula of semi-solid foods recommended in the PIN program was based on guidelines of complementary foods for Thai infant and toddlers by Thai Ministry of Public Health [36]. In the PIN program, Thai foods such as a pulp of Thai rice, pumpkin, egg yolk, some kinds of Thai vegetable, such as Coccinia grandis (dtam-leung) were recommended to the infants from 6 to 9 months after birth. In addition, half a teaspoon of vegetable oil per 250 g of semisolid foods was added to prevent fat soluble vitamin deficiencies [36].

#### 2.4.3. Biochemical Blood Tests and Nutrient Supplementation

Biochemical blood profiles were also assessed monthly as a high-risk follow-up clinic. One mL of blood sample was taken at blood drawing unit from all infants and hematocrit (Hct), calcium (Ca), phosphorus (P), alkaline phosphatase (ALP), and blood urea nitrogen (BUN) were examined in order to monitor iron deficiency anemia [37], bone health [38], and nutritional status, including sufficiency of protein. All analyses were conducted at the laboratory unit in the hospital. However, the historical group did not conduct a regular biochemical blood tests after discharge at the follow-up clinic visit. As a result, with an exception of Hct, a nutritional status of historical group was unable to be compared with the intervention group during 1- to 6-moCA and therefore did not include in the results of the current study. Moreover, monthly home visits were performed to monitor HM fortification procedure and infant feeding practices [19].

All infants received 2 mg/kg/day of iron at discharge. When abnormal levels of biochemical markers were identified, special nutrient supplementations were prescribed by a pediatrician at a follow-up clinic as these follows:

- Hct < 35%: Increase dose of ferrous sulfate/fumarate solution from 2 to 4–5 mg/kg/day [17,37].
- Total Ca < 8.0 mg/dL: Supplement calcium suspension 100–140 mg/kg/day [15,17]
- Phosphorus < 5.5 mg/day and/or ALP > 450 IU/L: Supplement phosphate solution 60–90 mg/kg/day [15,17]
- BUN > 20 mg/dL: Consider substitution with unfortified HM and/or standard term formula (TF) [10].

The infants also received 0.5 mL/day of multivitamin drop (MVD) (1 mL of MVD—composed of 2000 international unit (IU) of vitamin A, 400 IU of vitamin D and other water soluble vitamins)—until infants received total daily milk intake of >32 oz (1000 mL) [15]. However, non-fortified HM and/or TF was substituted when infants cannot tolerate with fortified HM and/or EF, or had an excessive rate of weight gain, and also showed Ca, P, and BUN above their normal levels [10].

Estimated volume of breastfeeding was derived by test weighing procedure [21]. Energy and selected nutrients intakes (protein, vitamin A and D, calcium, phosphorus, zinc, and iron), at 2-, 4-, and 6-moCA were estimated using HM composition values from literatures [39,40] manufacturer label claims for PF, EF and TF, drug label of MVD and ferrous fumarate drop, commercial complementary feeding formula and complementary foods composition recommended by the Thai Ministry of Public Health [36].

A nutritionist explained to the mothers/caregiver about the procedure of recording actual amount of all milk feeding, complementary food intake, and nutrient supplementation before infant discharge and also re-checked their practices monthly at the follow-up clinic visit. These records were kept by mothers/caregivers at home and by a nutritionist during home visit and at a follow-up clinic visit.

### 2.5. The Conventional Nutrition Services Conducted for the Comparison Group

Prior to their infant discharge, parents in the comparison group received general education such as latching technique and observations of feeding intolerance without specific nutritional education. After discharge, infants received unfortified HM and/or TF. They were provided with 2–5 mg/kg/day of ferrous fumarate solution and 0.5–1 mL/day of MVD according to a decision of the in-charge pediatrician at a follow-up clinic. Hematocrit was also tested for all infants at every clinic visits; while the other chemical blood tests, including Ca, P, ALP and BUN were performed at some visits according to pediatricians’ decisions.

Enteral intake data of the comparison group was also obtained from dietary intake recorded by mothers/caregivers and nutritional records by a nutritionist at a follow-up clinic as performed in the intervention group. Differences of the nutritional activities between the groups are described in Table 1.

### 2.6. Differences in Milk Formula between the Groups

The energy and nutrients values of fortified HM and EF used in the intervention group and unfortified HM [39,40] and TF used in the comparison group are shown in Table 2. Brands of TF formula were derived from nutritional records at a follow-up clinic in the historical group. Composition of TF is obtained from the average amount of ingredients from four commercial brands of TF in Thailand (S-26, Dumex Hi-Q, Lactogen and Enfalac A^+^) which contains equal energy density and protein per 100 mL, but are slightly different for the remaining nutrients.

The energy density of fortified HM and reconstituted formula of EF is 21.8 and 22.5 kcal/oz; while those of unfortified HM and TF is 19.7 and 20.1 kcal/oz, respectively. Fortified HM contains higher protein than unfortified HM and TF by 12.5% and 22.5%, respectively, but the remaining nutrients are equal to or less than TF. EF also contains higher protein, calcium, phosphorus, vitamin D and zinc by 42.9%, 79.2%, 70.6%, 33.8%, and 20.0%, respectively, and >100% of vitamin A and iron compared to those in TF.

The differences of feeding type and nutrient supplementation between the groups are summarized in Table 3.

### 2.7. Growth Assessments Conducted with Both Groups

Body weight, length and head circumference of infants in both groups were measured monthly, using revised anthropometric protocol for newborn by the same staff at the follow-up clinic. The hospital staff who perform growth measurement was trained and validated their measuring techniques every three months by the supervisor who is an expert in infant growth measurement. A digital balance (MS 2400; two decimals, maximum load 20 kg, maximum length measured 80 cm) was used for both weight and length measurements and a tape measure (Hochestmass 1.9 × 150 mm) was used for a head circumference measurement.

All data of the infants in the comparison group were recorded from patient charts during hospitalization and out-patient profiles at a follow-up clinic as secondary data until infants reached 6-moCA. Infant growth status was assessed by the Fenton [41] and WHO growth charts [42,43].

### 2.8. Statistical Analysis

Differences in energy and nutrient intakes between the groups were assessed by the Mann-Whitney *U* test. Comparisons of anthropometric variables between the two groups from 1- to 6-moCA were examined using multivariate of covariance (MANOVA) repeated measures. Mixed model analysis was used to adjust multiple measurements of each infant. The categorical data were compared using chi-squared test. Data analyses were conducted using PASW SPSS statistics (version 18.0, SPSS Inc., Chicago, IL, USA). All statistical analyses were examined with a significance level of 0.05.

## 3. Results

### 3.1. Baseline Characteristics of the Participants

Descriptive characteristics of infants were shown in Table 4. There were no statistically significant differences in baseline characteristics between the groups.

### 3.2. Human Milk Feeding and Introduction of Semi-Solid Foods

The volume of HM consumed (mL/kg/day) and proportion of HM volume per total volume of all milk feeds per day (%) among infants in the intervention group was greater than those of the comparison group (*p* < 0.05) at 6-moCA, but not 2-and 4-moCA. However, the total duration of HM feeding did not differ significantly between two groups. The length of time from birth to the introduction of semi-solids was also not significantly different (Table 5).

### 3.3. Nutritional Intake

Table 6 shows differences in daily energy and nutrient intakes of both groups. The intervention group showed greater energy and nutrient intakes except in zinc and iron compared to the comparison group at 2-, 4- and 6-moCA. It was also found that a median iron intake in both groups were slightly greater than the daily recommendation for healthy term infants.

### 3.4. Infant Growth Outcomes

**Figure 2:**
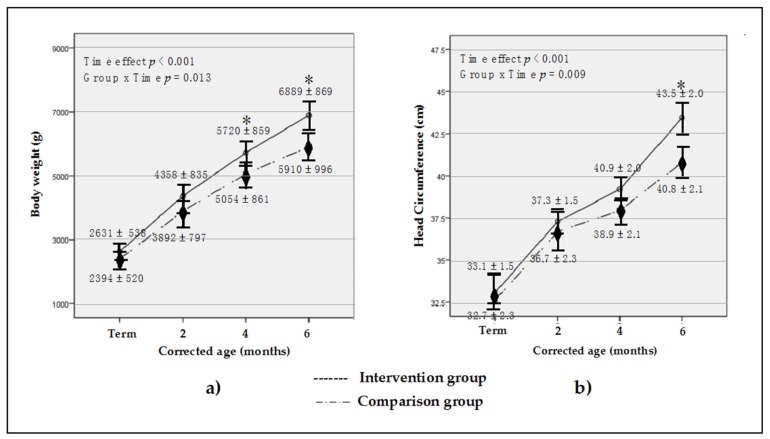
Anthropometric measurement of body weight, (**a**) and head circumference, (**b**) of the intervention and comparison group (*n* = 22). Data are reported as unadjusted mean ± SD. * denote a significant difference between groups at a specific time point with intervention greater than the comparison group (body weight at 4-moCA, *p* = 0.001, and 6-moCA, *p* < 0.001; head circumference at 6-moCA, *p* < 0.001).

Compared to the comparison group, the intervention group showed significantly greater body weight (*p* = 0.013) at 4- and 6-moCA and head circumference (*p* = 0.009) at 6-moCA (Figure 2). However, no significant difference in length and weight gain velocity were observed between two groups at any time point of age. Furthermore, a greater proportion of the intervention group reached body weight greater than −2SD (*Z* score) at 4-moCA (81.8% vs. 45.5%, *p* = 0.027) and 6-moCA (90.9% vs. 50.0%, *p* = 0.007) compared with their comparison counterpart. In addition, the intervention group reached head circumference greater than −2SD (*Z* score) at 6-moCA (95.5% vs. 54.5%, *p* = 0.004) compared to the comparison group (Table 7). On the other hand, no differences were observed for length. Also, there was no over-standard growth or growth with higher +2SD (*Z* score) for body weight, length, and head circumference in both groups. Moreover, there was no correlation between HM feeding duration and any growth outcomes.

## 4. Discussion

The current study found an increase in the proportion of infants who were underweight (after adjustment for gestational age) in both groups; 4.5% at birth and 86.3% at discharge for the intervention group and 9.1% at birth and 86.9% at discharge for the comparison group. The results may indicate that early protein and energy deficits were not recovered during hospital admission regardless of the groups. Our findings were consistent with previous studies [4,8,9,44,45] and the present study suggested a difficulty of preventing SGA among VLBW preterm infants during hospital admission.

The present study showed differences in anthropometric variables of fortified HM and/or EF fed versus HM alone and/or TF fed VLBW preterm infants from discharge to 6-moCA. The results indicated that the intervention group had significantly greater weight at 4- and 6-moCA and head circumference at 6-moCA compared to the comparison group. These results indicated the usefulness of the PIN program to compensate for energy and nutritional deficits which VLBW preterm infants experienced during their hospital stay and better recovery using fortified HM and/or EF. While there are controversies in beneficial effects of both fortified HM and enriched formula on growth parameters in preterm infants at post-discharge [7,18,46], the results from the current study were consistent with other studies that observed better recovery in infants who received energy and nutrient-enriched HM or formula at early hospital discharge [47,48,49,50].

In addition, the results showed significant differences in a proportion of infants reaching standard weight at 4- and 6-moCA, and head circumference at 6-moCA while no difference was observed at 2-moCA. This result showed that the PIN program may be effective in shortening the period of post-discharge growth restriction by 4-moCA without rapid acute effects. Casey, et al. [45] reported that achieving optimal catch-up growth during a post-natal period, especially by six months after term, will significantly result in better neurodevelopmental outcomes from 18 months to 6 years compared with those who achieved catch-up growth later. Considering suggestion by Casey, et al. [45], the PIN program that provides adequate nutrition to preterm infants during such a critical period may have beneficial impact on both short- and long-term health in this particular population.

Consistently, the nutrient intakes of infants with fortified HM and EF during study period were greater and more consistent with current dietary recommendations [15,23] than infants with unfortified HM and/or TF. These findings may attribute to the effect of enriching HM and formula to increase energy and protein intake. In addition, MVD, Ca, and P supplementation prescribed at a follow-up clinic according to the study protocol resulted in significantly higher intake of these nutrients. However, iron and zinc intakes did not differ between groups. This may be because of a similarity in iron and zinc supplementation practices in both groups. The results also showed a greater volume of human milk and the proportion of feed volume in the intervention group at 6-moCA. However, based on the obtained results, we interpreted the results as due to higher consumption of energy and protein that contained in enriched HM rather than increased volume.

While the PIN program included an education for mothers about benefits of breastfeeding before infant discharge, the present study did not show improvement in feeding duration between the groups and, in fact, was shorter than a previous study that reported a proportion and duration of exclusive HM feeding [20]. The results may indicate insufficient and inadequate lactation support of the PIN program to overcome problems that mothers experienced at post-discharge. Although the PIN program made more frequent home visits compared to the conventional service, the content including educational tools delivered prior to discharge and at each home visit should be adjusted as well as increasing involvement of lactation experts and frequencies of both on-site and distant counseling opportunities. In addition, the current study may indicate a presence of additional issues that prevent mothers from continuing HM feeding after discharge. As a result, further research may be warranted to evaluate factors that influence mothers to continue feeding HM to their child.

### Study Limitations

Limitations of the current study included using the historical comparison group which contributed to slightly to differences in composition of TF formula intake. In addition, some data deviations might occur, such as an estimation of breast milk volume intake in some breastfed infants due to inadequate skill of test-weighing procedure in some mothers. Next, the currently applied inclusion and exclusion criteria also limited the extrapolation of the observations as documented in this cohort to all cases. Furthermore, except Hct, we cannot compare the nutritional status of biochemical blood test from 1- to 6-moCA. The main reason is that for the historical group, there were no regular biochemical blood tests after discharge at the follow-up clinic visit in the previous routine practice. The small sample size limits the interpretation and generalization of these results. Further studies are recommended in this area. Lastly, we use non-randomization to evaluate the program, resulting from a limitation of number of participants in a research setting.

## 5. Conclusions

Enlistment in a PIN program with enriched HM and formula feeding from hospital discharge to 6-moCA resulted in significantly reduced post-discharge growth restrictions for VLBW preterm babies compared to those who received unfortified HM and TF in conventional practices. Regular monitoring is needed to educate mothers or caregivers not to under- or overfeed their infants. Further lactation support and developed maternal education programs are needed to encourage continued HM feeding after discharge. Further studies are warranted with concurrent comparison groups and a longer follow-up period to analyze infant growth, neurodevelopment, and body composition.

## Figures and Tables

**Table 1:** Comparisons of activities in the preterm infant post-discharge nutrition (PIN) program and conventional nutrition services.

The PIN Program Activities	Conventional Nutrition Activities
**I. Pre-discharge activities**	
Giving formal nutritional education to parents/caregivers	No formal nutritional education for parents/caregivers
**II. Post-discharge activities**	
Giving enriched-nutrient HM and/or EF to the infants from discharge to 6-moCA	Giving unfortified HM and/or TF from discharge to infant weaning
Using revised biochemical blood tests and nutrition supplements protocol	Using biochemical blood tests according to decisions by in-charge pediatricians
Using revised criteria to start and recommended types of semi-solid foods to preterm infants	Suggestions mainly about time to start semi-solid foods at six months after birth without other specific criteria to start semi-solid foods
Monthly home visit until preterm infants reached 6-moCA	Home visit only one time after hospital discharge

moCA = months corrected age; HM = human milk; EF = enriched-nutrient formula; TF = term formula.

**Table 2:** Composition of energy and selected nutrients in fortified human milk (HM) and enriched-nutrient formula (EF) in the intervention group versus unfortified HM and term formula (TF) in the comparison group.

Composition	Intervention Group		Comparison Group	
	Fortified HM	EF	Unfortified HM	TF
Energy (kcal)	72.60	75.00	65.60	67.00
Protein (g)	1.80	2.10	1.60	1.47
Vitamin A (IU)	122.40	343.30	72.00	160.83
Vitamin D (IU)	20.50	63.00	12.00	47.07
Calcium (mg)	38.03	82.00	29.00	45.77
Phosphorus (mg)	19.03	48.00	14.00	28.13
Zinc (mg)	0.63	0.72	0.53	0.60
Iron (mg)	0.25	1.37	0.13	0.63

HM = human milk, Fortified HM = expressed HM + preterm formula (PF: as a human milk fortifier), EF = enriched formula, TF = term formula, Milk composition is per 100 mL as provided by the literature and manufacturer, EF and TF were available cow milk protein-based formula.

**Table 3:** Comparisons of feeding type and nutrient supplementations for preterm infants after discharge between the intervention and comparison group.

	Intervention Group	Comparison Group
**I. Feeding type**		
1.1 Milk intake at discharge to 6-moCA	Fortified HM and/or EF with energy density of 22 kcal/oz	Unfortified HM and/or TF with energy density of 20 kcal/oz
1.2 Semi-solid foods	Recommended formula based on complementary food guideline for infants and toddlers by Thai MOPH	Commercial formula and/or any formula based on caregivers’ decision
**II. Nutrient supplementations**		
2.1 At discharge		
Iron	2 mg/kg/day, increase to 4–5 mg/kg/day if Hct < 35%	2–5 mg/kg/day *
MVD	0.5 mL/day until infants had total daily milk intake of >32 oz	0.5–1 mL/kg/day *
Calcium suspension	100–140 mg/kg/day if total Ca < 8.0 mg/dL	90–120 mg/kg/day *
Phosphate solution	60–90 mg/kg/day if P < 5.5 mg/day and/or ALP > 450 IU/L	60–90 mg/kg/day *

* refers to dose of nutrient supplementation based on pediatricians’ decisions, HM = human milk, EF = enriched formula, TF = term formula, moCA = months corrected age, MOPH = Ministry of Public Health, Hct = hematocrit, MVD = multivitamin drop, Ca = calcium, dL = deciliter, P = phosphorus, ALP = alkaline phosphatase.

**Table 4:** Baseline characteristics of the intervention and comparison group.

Variables	Intervention Group (*n* = 22)	Comparison Group (*n* = 22)	*p*-Value
Sex, *n* (%)	Male, 13 (59.0)	Male, 11 (50.0)	0.763
GA at birth, week ± day	29 ± 12 day	30 ± 14 day	0.501
Twin, *n* (%)	3 (13.6)	6 (27.3)	0.457
Growth status at birth			
Birth weight, g	1192 ± 202	1151 ± 208	0.502
Length, cm	39.1 ± 2.6	38.4 ± 3.7	0.525
Head circumference, cm	26.1 ± 1.8	26.4 ± 1.9	0.626
SGA at birth			
Body weight, *n* (%)	1 (4.5)	2 (9.1)	1.000
Length, *n* (%)	1 (4.5)	1 (4.5)	1.000
Head circumference, *n* (%)	2 (9.1)	2 (9.1)	1.000
SGA at discharge			
Body weight, *n* (%)	19 (86.4)	20 (90.9)	1.000
Length, *n* (%)	10 (45.5)	14 (63.6)	0.364
Head circumference, *n* (%)	15 (68.2)	12 (54.5)	0.453
PMA at discharge, week ± day	37 ± 12 day	38 ± 20 day	0.199
LOS, days	52 ± 12	53 ± 17	0.895
Feeding type at discharge, *n* (%)			
Breast milk feeding	18 (81.8)	17 (77.3)	1.000
Mixed feeding	3 (13.6)	4 (18.2)	1.000
Standard formula	1 (4.5)	2 (9.1)	1.000
Growth status at term (40 weeks)			
Body weight, g	2631 ± 538	2426 ± 531	0.145
Length, cm	47.7 ± 2.7	46.9 ± 2.9	0.318
Head circumference, cm	33.1 ± 1.6	32.2 ± 2.3	0.470
Maternal age (years)	22.5 ± 4.8	25.3 ± 5.5	0.087

*n* = number of infant; GA = gestational Age; SGA = small of gestational age; PMA = postmenstrual age; LOS = length of hospital stay. Data are expressed as mean ± SD. The differences between the groups were determined by chi-squared test.

**Table 5:** Comparisons of exclusively human milk feeding, feeding duration and a timing of introducing semisolid foods between the groups.

	Intervention Group (*n* = 22) Median (Q1–Q3)	Comparison Group (*n* = 22) Median (Q1–Q3)	*p*-Value
Exclusively of HM feeding			
2-moCA			
HM intake, mL/kg/day	40.37 (5.51–111.14)	61.20 (3.05–139.55)	0.234
Proportion of HM volume per	35.50 (10.17–79.17)	48.50 (20.21–120.21)	0.381
Total volume of milk feeds per day, %			
4-moCA			
HM intake, mL/kg/day	24.67 (13.34–61.55)	27.45 (10.00–57.80)	0.761
Proportion of HM volume per	22.82 (15.09–61.09)	21.86 (16.33–56.33)	0.859
Total volume of milk feeds per day, %			
6-moCA			
HM intake, mL/kg/day	11.73 (3.16–13.51)	3.05 (1.12–11.43)	0.044 *
Proportion of HM volume per	11.82 (4.43–16.43)	2.27 (0.56–7.94)	0.041 *
Total volume of milk feeds per day, %			
Total HM feeding duration, weeks after birth	16.00 (10.00–26.00)	18.00 (12.00–24.00)	0.427
Time at introduction of semisolid foods, weeks after birth	20.00 (17.00–23.00)	21.00 (19.00–24.00)	0.625

*n* = number of infant, Q1 = the first quartile, Q3 = the third quartile, HM = human milk. moCA = months corrected age. Total HM feeding duration refers to duration of exclusively HM feeding and/or mixed feeding (HM feedings plus formula feedings). Differences between groups were assessed by Mann-Whitney *U* test. * refers to statistically significant differences.

**Table 6:** Comparisons of daily energy and nutrient intakes of preterm infants between the groups.

Energy/Nutrient	Intervention Group (*n* = 22) Median (Q1–Q3)	Comparison Group (*n* = 22) Median (Q1–Q3)	*p*-Value	RDA
**Energy, kcal/kg**				
2-moCA	91.08 (89.27–93.46)	88.40 (85.85–91.74)	0.014	85–120
4-moCA	86.39 (82.15–88.22)	76.67 (72.65–78.16)	0.001	
6-moCA	82.07 (80.11–88.63)	69.22 (66.49–71.98)	<0.001	
**Protein, g/kg**				
2-moCA	2.87 (2.44–3.30)	2.21 (1.95–2.69)	0.024	1.5–2.5
4-moCA	2.48 (2.01–2.89)	2.07 (1.73–2.17)	0.037	
6-moCA	1.85 (1.67–2.25)	1.53 (1.39–1.63)	<0.001	
**Vitamin A, IU/kg**				
2-moCA	555.66 (294.81–779.81)	219.66 (165.33–357.06)	0.004	320–700
4-moCA	476.99 (301.20–665.91)	209.63 (185.04–238.95)	<0.001	
6-moCA	389.03 (227.10–521.58)	202.24 (191.11–221.02)	0.035	
**Vitamin D, IU/kg**				
2-moCA	107.98 (45.14–145.78)	50.00 (36.09–71.00)	0.007	60–160
4-moCA	91.19 (55.00–125.30)	42.72 (32.06–47.84)	<0.001	
6-moCA	73.23 (40.22–96.57)	40.78 (38.40–44.72)	<0.001	
**Calcium, mg/kg**				
2-moCA	85.80 (63.95–88.95)	68.24 (63.17–69.86)	<0.001	60–120
4-moCA	94.64 (78.61–100.08)	54.20 (51.76–57.94)	<0.001	
6-moCA	66.01 (49.04–69.60)	48.75 (45.77–49.87)	0.016	
**Phosphorus, mg/kg**				
2-moCA	40.43 (31.31–59.31)	33.13 (30.00–36.92)	<0.001	35–60
4-moCA	71.31 (66.32–77.83)	32.29 (30.14–34.73)	<0.001	
6-moCA	44.35 (35.08–48.81)	30.30 (29.66–34.33)	0.018	
**Zinc, mg/kg**				
2-moCA	0.78 (0.70–0.84)	0.74 (0.70–0.78)	0.226	0.5–1
4-moCA	0.72 (0.64–0.79)	0.71 0.69–0.75)	0.451	
6-moCA	0.67 (0.59–0.72)	0.64 (0.59–0.69)	0.572	
**Iron, mg/kg**				
2-moCA	4.95 (4.30–5.72)	4.37 (3.99–5.16)	0.080	2–4
4-moCA	5.02 (3.64–5.47)	5.30 (4.16–5.49)	0.411	
6-moCA	4.73 (3.17–5.58)	4.51 (2.68–5.41)	0.231	

Q1 = the first quartile, Q3 = the third quartile, PIs = preterm infants, RDA = recommended dietary allowance, the upper limit is for PIs 37–40 weeks GA, the lower limit is for term infants at six months of age, no = number, moCA = months corrected age, IU = international unit.

**Table 7:** Comparisons of growth status of preterm infants between the groups.

	*n* (%)		
Growth Status	Intervention Group (*n* = 22)	Comparison Group (*n* = 22)	*p*-Value
2-moCA, higher−2SD (*Z* score) in			
Body weight	14 (63.6)	9 (40.9)	0.227
Length	15 (68.2)	8 (36.4)	0.069
Head circumference	17 (77.3)	10 (45.5)	0.062
4-moCA, higher−2SD (*Z* score) in			
Body weight	18 (81.8)	10 (45.5)	0.027 *
Length, *n* (%)	17 (77.3)	10 (45.5)	0.062
Head circumference, *n* (%)	18 (81.8)	11 (50.0)	0.055
6-moCA, higher−2SD (*Z* score) in			
Body weight	20 (90.9)	11 (50.0)	0.007 *
Length	17 (77.3)	11 (50.0)	0.116
Head circumference	21 (95.5)	12 (54.5)	0.004 *

moCA = months corrected age, −2SD (*Z* score) = −2SD line in *Z* score growth chart for newborn by World Health Organization. Data are expressed as number of cases (percentage). The difference between intervention and comparison group was determined by chi-square test. * refers to statistical significance.
